# Pharmacological management of seizures in patients with COVID-19: a systematic review

**DOI:** 10.12688/aasopenres.13224.2

**Published:** 2021-11-23

**Authors:** Priscilla Kolibea Mante, Nana Ofori Adomako, John-Paul Omuojine, Paulina Antwi

**Affiliations:** 1 Department of Pharmacology, Kwame Nkrumah University of Science and Technology, Kumasi, Ghana; 2 Department of Pharmacy Practice, Kwame Nkrumah University of Science and Technology, Kumasi, Ghana; 3 Department of Psychiatry, Komfo Anokye Teaching Hospital, Kumasi, Ghana

**Keywords:** SARS-CoV-2, neurological symptoms, levetiracetam, status epilepticus, epilepsy

## Abstract

**Background: **Some patients with severe acute respiratory syndrome coronavirus 2 (SARS-CoV-2) have been reported to exhibit neurological symptoms such as seizures and impaired consciousness. Our study reviews reported cases to assess the pharmacological approach to managing seizures in SARS-CoV-2 patients and associated outcomes.

**Methods: **A systematic review of case reports on the incidence of seizures following coronavirus disease 2019 (COVID-19) among patients that reported use of antiepileptic drugs (AEDs) in management was performed by using the PRISMA (preferred reporting items for systematic reviews and meta-analysis) guidelines. Databases used included EMBASE, PubMed, SCOPUS, and Google Scholar. Data was presented as qualitative and descriptive data.

**Results: **In total, 67 articles were selected for full-text assessment, of which 18 were included in the final review. Patients had a median age of 54 years, most of whom were male. Remdisivir, dexamethasone, Laninamivir, hydroxychloroquine, azithromycin, and Lopinavir-ritonavir were common agents used in the management of COVID-19. Most patients presented with either generalized tonic-clonic seizures or status epilepticus. Most patients received levetiracetam as drug choice or as part of their regimen. Other AEDs commonly prescribed included midazolam and sodium valproate. Some patients received no antiepileptic drug therapy. Most of the patients who died had more than one comorbidity. Also, most of the patients who died received COVID-19 treatment drugs. None of the patients who received midazolam as drug choice or as part of their regimen developed recurrent seizures in contrast to patients who received levetiracetam and sodium valproate as drug choice or as part of their regimen. Interestingly, none of the patients who received no AEDs suffered recurrent seizures or died.

**Conclusions: **Standard guidelines for managing seizures in COVID-19 patients may be required. A limitation of this review is that it involved the use of case reports with no controls and a small number of patients.

## Introduction

Neurological manifestations have been reported in about one-third of patients with coronavirus disease 2019 (COVID-19). In addition to the primary respiratory symptoms, there have been neurological symptoms manifested at all levels of the nervous system ( Mao *et al.*, 2020). Several case studies on severe acute respiratory syndrome coronavirus 2 (SARS-CoV-2) infection have demonstrated neurological effects such as strokes, loss of consciousness, encephalopathy, generalized tonic-clonic convulsions and neuralgia ( Beyls *et al.*, 2020). This is not entirely surprising as SARS-CoV-1 and Middle East respiratory syndrome coronavirus (MERS-CoV) have previously been associated with sporadic neuropathological changes ( Desforges *et al.*, 2020; Lau *et al.*, 2004).

SARS-CoV-2 has been proposed to enter the central nervous system (CNS) either via systemic vascular dissemination or by crossing the cribriform plate of the ethmoid bone ( Baig *et al.*, 2020). The latter mechanism is speculated to contribute to anosmia experienced by a quarter of patients ( Baig *et al.*, 2020). Similar to SARS-CoV-1, the new SARS-CoV-2 is believed to make its way into biological cells via the angiotensin-converting enzyme 2 (ACE2) receptor ( Lan *et al.*, 2020).

Although few cases have been reported, studies indicate that seizures may occur early in the disease process. Nevertheless, the presence of seizures bears heavily on the management and outcome of patients with SARS CoV-2. Recurrent or prolonged seizures, as occurs in status epilepticus, may contribute to or worsen hypoxic encephalopathy, cerebrovascular events, and cytokine storms that can further lead to acute seizures ( Bartiromo *et al.*, 2020).

Further to this, COVID-19 may be more difficult to treat in patients exhibiting seizures than in other patients. Antiepileptic drugs (AEDs) tend to cause complex drug-drug interactions and adjustment of these drugs may be necessary to prevent heart, liver, or kidney complications that may occur in patients with severe COVID-19 ( Asadi-Pooya *et al.*, 2020). No special guidelines currently exist for management of seizure symptoms in COVID-19. Hence, seizures in patients are controlled with currently available AEDs based on seizure types exhibited by patients. We provide a review of publications on cases of seizure disorders reported in patients with COVID-19, the pharmacological approach to management and outcomes on mortality exploring implications on management of patients. In the wake of a pandemic that has prompted experimental clinical therapy, reviews of the prevailing practices may provide information to guide clinical decision-making. Our objective was therefore to assess which antiepileptic medication or medications are most effective for managing seizures occurring in patients with COVID-19.

## Methods

### Strategy for literature search

The Preferred Reporting Items for Systematic Reviews and Meta-Analyses (PRISMA) guidelines were used to perform a systematic review of the literature ( Moher *et al.*, 2010). Comprehensive electronic literature searches were performed in EMBASE, PubMed, SCOPUS, and Google Scholar to identify articles that covered the neuropathological symptoms in COVID-19. This review primarily aimed to synthesize information on seizure-related complications in patients with COVID-19.

The search included articles published in the English Language from January to December, 2020. Keywords used included ‘ *neurology*’ or ‘ *neurological manifestations*’ or ‘ *nervous system*’, ‘ *neuropathy’* or ‘ *nerves*’ and ‘ *COVID-19*’ or *‘SARS-CoV-2’* and *‘antiepileptic drugs’* or ‘ *antiseizure drugs’*. A combination of keywords and MeSH terms were applied to maximize the output from literature findings. The full search string for the database search was COVID-19* OR SARS-CoV-2* OR AND neurological manifestations* OR nervous system* AND antiepileptic drugs* OR antiseizure drugs*. The bibliography list of selected articles were screened by reading through the corresponding abstracts in order to identify additional appropriate studies. Screening was done by JPO and disagreements were resolved by all authors. As seizure complications were rare in COVID-19, all articles reporting seizures were included. We included all types of articles or preprints (from the bioRxiv) that met the following criteria: reported the incidence of seizures following COVID-19 among patients of all ages or reported the use of antiepileptic drugs (AEDs) in the management of COVID-19-related seizures. Studies that reported on patients with a history of seizure disorders were excluded from the study. Only reported cases of seizure complications for which explicit temporal or causal association with COVID-19 infection could be determined were included in the review. Animal studies were excluded. All studies selected were initially managed using Microsoft Word v16.45. This review has not been registered and a review protocol was not prepared.

### Data extraction

Data was extracted with the aid of a pre-designed data extraction form. Data extraction from full text of eligible articles was done independently by two investigators (PKM and NOA). The following data were obtained: author, country of report, demographic details, the number of patients with COVID-19 having seizure complications, frequency and prevalence of seizures, electroencephalogram (EEG), neuroimaging and/or other laboratory investigations associated with other neuropathological symptoms, management strategies and outcomes. Accuracy of extracted data was rechecked by a third independent investigator. Investigators made every effort to prevent data duplication. Quality of the included cases was assessed based on Consensus-based Clinical Case Reporting (CARE) guidelines for case reports by ensuring the selected cases met the criteria as stated in the 2013 CARE checklist.

### Data synthesis and statistical analyses

Studies were first tabulated as qualitative data as selected studies were case reports. Given the small number of cases, descriptive analysis was performed to generate frequencies and percentages. All analyses and data visualisation were conducted using STATA version 13 (StataCorp, College Station, TX, USA).

## Results

### Study characteristics

**Figure 1.**
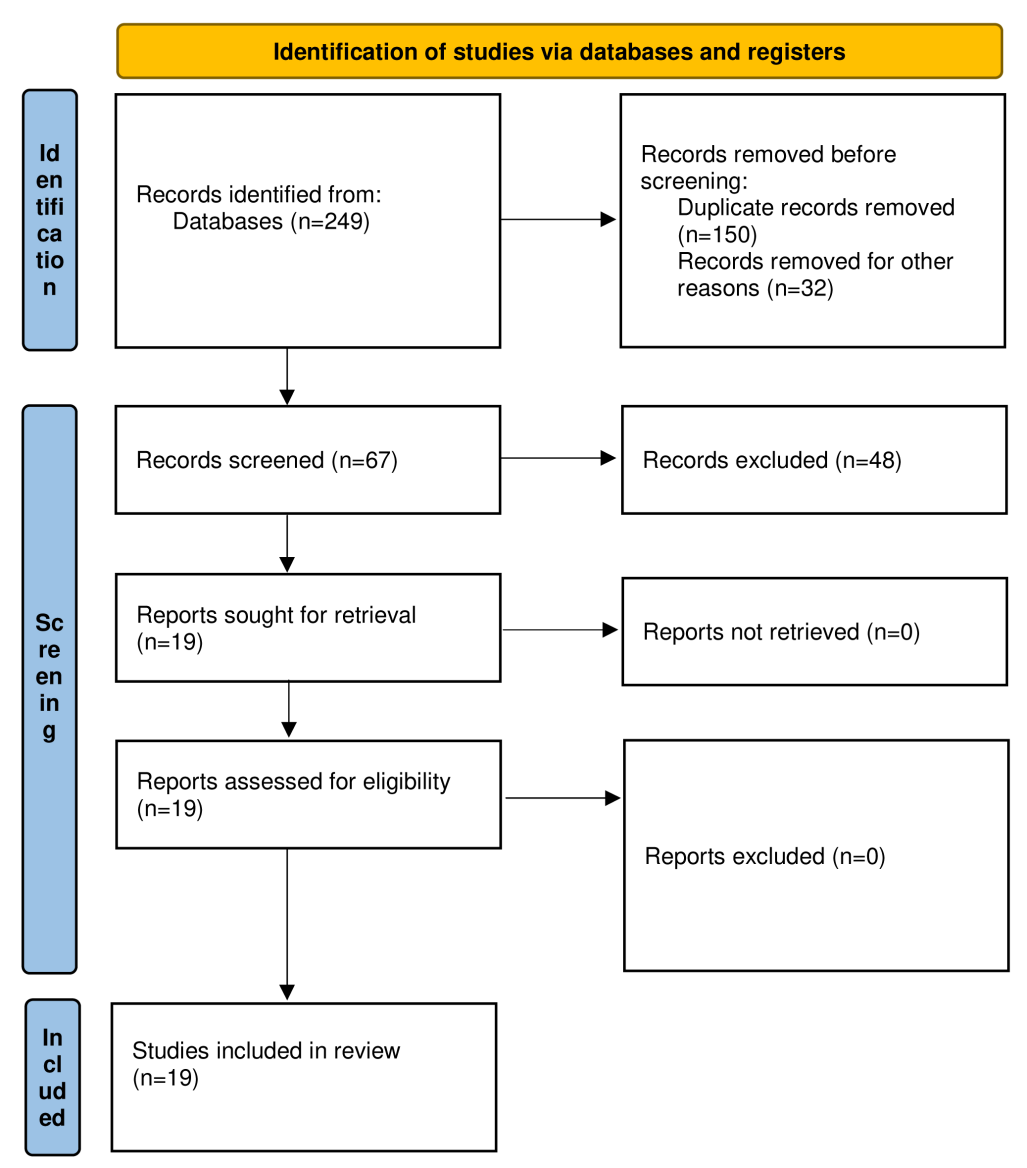
Preferred Reporting Items for Systematic reviews and Meta-Analyses (PRISMA) 2020 flow diagram of the literature search and studies included in review ( Page *et al.*, 2021).

A search of literature yielded 249 citations. Following duplicates removal, and titles and screening of abstracts, 67 articles were selected for full-text assessment. Subsequent to full-text evaluation, 18 articles were used for the final review ( Figure 1). Characteristics of the included articles are provided in Table 1. All articles were case reports/series. One article was a multicenter study. Three case reports were of fair quality whereas all other studies were graded to be of good or excellent quality according to the Murad tool for methodological quality and risk of bias. Studies were considered as “poor,” “moderate/fair,” or “good/excellent” quality when 3 or fewer, 4, or 5 of the criteria were fulfilled, respectively ( Murad *et al*., 2018).

**Table 1.** Descriptive characteristics of included studies.

Reference	Patient No.	Country	COVID-19 Drugs	AEDs	Sex	Age (yrs.)	Seizure Disorder Reported	Comorbidities	Seizure History	Outcome
**( Karambelkar *et al.*, 2020)**	1	USA	Hydroxychloroquine.	Not stated	male	32	Generalized Tonic-Clonic Seizure	Sleep apnea	Negative	Patient ultimately discharged from intensive care unit after being deemed hemodynamically stable.
	2	USA	None	Lorazepam, Levetiracetam	male	82	Generalized Tonic-Clonic Seizure	Chronic systolic heart failure, atrial fibrillation, hypertension	Negative	The patient succumbed to septic shock despite optimal medical therapy.
**( Abdulsalam *et al.*, 2020)**	3	Kuwait	Lopinavir-Ritonavir, Hydroxychloroquine	Diazepam, Midazolam, Levetiracetam	male	32	Generalized Tonic-Clonic Status Epilepticus	Nil of note	Negative	Convulsions aborted on Midazolam. He was discharged after 14 days in stable condition, remaining afebrile after two negative swabs for SARS-CoV-2.
**( Balloy *et al.*, 2020)**	4	France	None	Clobazam, Levetiracetam	male	59	Non- convulsive status epilepticus	Atrial fibrillation, obstructive sleep apnea	Negative	Patient was clinically stable and discharged from ICU.
**( Chen *et al.*, 2020)**	5	USA	Remdisivir	Levetiracetam, Phenytoin	female	37	Status epilepticus	Multiple; end stage renal disease	Negative	Seizures resolved, discharges slowed, mental status improved.
	6	USA	Remdisivir	Levetiracetam	female	60	Non- convulsive status epilepticus	Hypertension	Negative	Improvement.
	7	USA	Remdisivir	Levetiracetam	male	50	Seizure-like events	Nil of note	Negative	Rapid-EEG showed moderate to severe seizure activity.
	8	USA	Remdisivir	Levetiracetam	female	38	Seizure-like events	Orthotopic heart transplant, heterotopic kidney transplant, diabetes, chronic congestive hepatopathy, pulmonary hypertension	Negative	No epileptiform discharges or seizures observed. Rapid-EEG converted to conventional EEG, showed moderate- severe.
**( Gómez-Enjuto *et al.*, 2020)**	9	Spain	Hydroxychloroquine, Lopinavir-ritonavir, dexamethasone, ceftriaxone, enoxaparin	Diazepam, lacosamide, sodium valproate	male	74	Refractory Status Epilepticus	IgG kappa multiple myeloma	Not stated	Patient stabilized.
**( Emami *et al.*, 2020)**	10	Iran	None	Levetiracetam, midazolam	female	54	Status Epilepticus	HIV	Negative	Expired in ICU.
	11	Iran	Hydroxychloroquine	Midazolam	male	42	Status Epilepticus	Nil of note	Negative	Expired in ICU.
	12	Iran	Lopinavir-Ritonavir	Midazolam, levetiracetam	male	35	Seizure-like events	HIV	Negative	Expired in ICU.
	13	Iran	Dexamethasone	Phenobarbital, levetiracetam, valproate, phenytoin, midazolam, thiopental, fentanyl	male	2.9	Seizure-like events	Nil of note	Negative	Discharged.
	14	Iran	None	Phenobarbital	female	2 days	Seizure-like events	Low birth weight	Negative	Expired in ICU.
**( Bhatta *et al.*, 2020)**	15	USA	None	Lorazepam, Levetiracetam	male	11	Generalized tonic-clonic seizure	Nil of note	Negative	Seizures aborted.
**( Moriguchi *et al.*, 2020)**	16	Japan	Laninamivir	Levetiracetam	male	24	Generalized tonic-clonic seizure	Nil of note	Negative	Patient was still undergoing treatment.
**( Duong *et al.*, 2020)**	17	USA	Hydroxychloroquine	Levetiracetam	female	41	Generalized tonic-clonic seizure	Obesity, Diabetes	Negative	Improvement in mentation.
**( Haddad *et al.*, 2020)**	18	USA	Hydroxychloroquine, azithromycin	None	male	41	Generalized tonic-clonic seizure	Well controlled HIV	Negative	On day 6 of hospitalization, the patient’s level of consciousness improved off sedation and was successfully extubated.
**( Sohal & Mansur, 2020)**	19	USA	Hydroxychloroquine, azithromycin	Levetiracetam, Valproate	male	72	Tonic seizures	Coronary artery disease with stent, DM type 2, Hypertension, end stage kidney disease	Negative	A code blue was called on admission day 5 after patient became pulseless. Return of Spontaneous Circulation (ROSC) was not be achieved.
**( Bernard-Valnet *et al.*, 2020)**	20	Switzerland	None	Clonazepam, Valproate	female	64	Focal status epilepticus	None stated	Negative	The patient markedly improved 96h after admission with resolution of her symptoms.
	21	Switzerland	None	None	female	67	Unclassified	None stated	Negative	Neurological symptoms resolved within 24h.
**( Zanin *et al.*, 2020)**	22	Italy	None	lacosamide, levetiracetam, phenytoin	female	54	Generalized tonic Clonic seizures	AcomA aneurysm	Negative	The patient was transferred to rehabilitation without sensorimotor deficits after 12 days.
**( Hepburn *et al.*, 2021)**	23	USA	Not stated	Levetiracetam	male	76	Myoclonic and Focal Seizures	Asthma, , chronic kidney disease, diastolic dysfunction, hypertension hyperlipidemia, left bundle branch block, cervical fusion	Negative	Clinical and electrographic seizure activity subsided.
	24	USA	Not stated	Levetiracetam	male	82	Focal Status Epilepticus	COPD, complete heart block, chronic kidney disease, venous thromboembolic disease,	Negative	Seizure frequency improved after receiving levetiracetam. Patient remained on the ventilator, family opted for withdrawal of life- sustaining support after 20 days of ICU stay
**( Logmin *et al.*, 2020)**	25	Germany	Not stated	Not stated	female	70	Non- epileptic seizures/ Convulsive syncope	Syncope, neuropathic pain, atrial fibrillation	Negative	She recovered well without requiring intensive care.
**( Fasano *et al.*, 2020)**	26	Italy	Lopinavir-Ritonavir	None	male	54	Focal Seizure	Nil of note	Negative	Patient’s EEG recorded the following day after first incident indicated no abnormalities. Patient recovered after 2 weeks of antiviral therapy
**( Gaughan *et al.*, 2020)**	27	Ireland	None	None	male	87	Generalized tonic-clonic seizure	Nil of note	Negative	Patient remained clinically well, no respiratory symptoms during admission. EEG was performed following discharge demonstrated intermittent fronto- temporal dysfunction maximal on the right side, compatible with the known imaging abnormalities.
	28	Ireland	None	Lorazepam	female	77	Generalized tonic-clonic seizure	Nil of note	Negative	Seizure aborted, Montreal Cognitive Assessment (MOCA) performed four months post hospitalization was 24/30, suggesting persistent cognitive deficit.
**( Elgamasy *et al.*, 2020)**	29	Germany	None	Levetiracetam, Clobazam, Lacosamide Magnesium	female	73	Focal seizure	Hypertension	Negative	The patient was discharged home for self‐isolation

### Patient characteristics

The 18 articles reported incidence of seizure complications among 29 patients with COVID-19 from ten countries (USA [n=12], Germany [n=2], Italy [n=2], Switzerland [n=2], Iran [n=5], Ireland [n=2], Spain [n=1], France n=1], Japan [n=1], and Kuwait [n=1]). Of these, 13 (44.8%) had presented with seizures as their primary complaint and first symptom of COVID-19. One study described the incidence of reversible posterior leukoencephalopathy syndrome (PRES) presenting as refractory status epilepticus. The age of the patients ranged from 2 days to 82 years, the median being 54 (interquartile range, 37–72) years. Of the patients, 41.4% (12/29) were females ( Table 1). Moreover, 37.9% (11/29), 34.5% (10/29), and 25.8% (8/29) had 0, 1, and ≥2 comorbidities, respectively. COVID-19 treatment drugs were reported for 52% (15/29) of patients. The treatments for COVID-19 received included remdisivir (4 patients), dexamethasone (1 patient), Laninamivir (1 patient), hydroxychloroquine (7 patients), azithromycin (1 patient), and Lopinavir-ritonavir (4 patients).

### Seizure types and antiepileptic drugs administered

**Figure 2.**
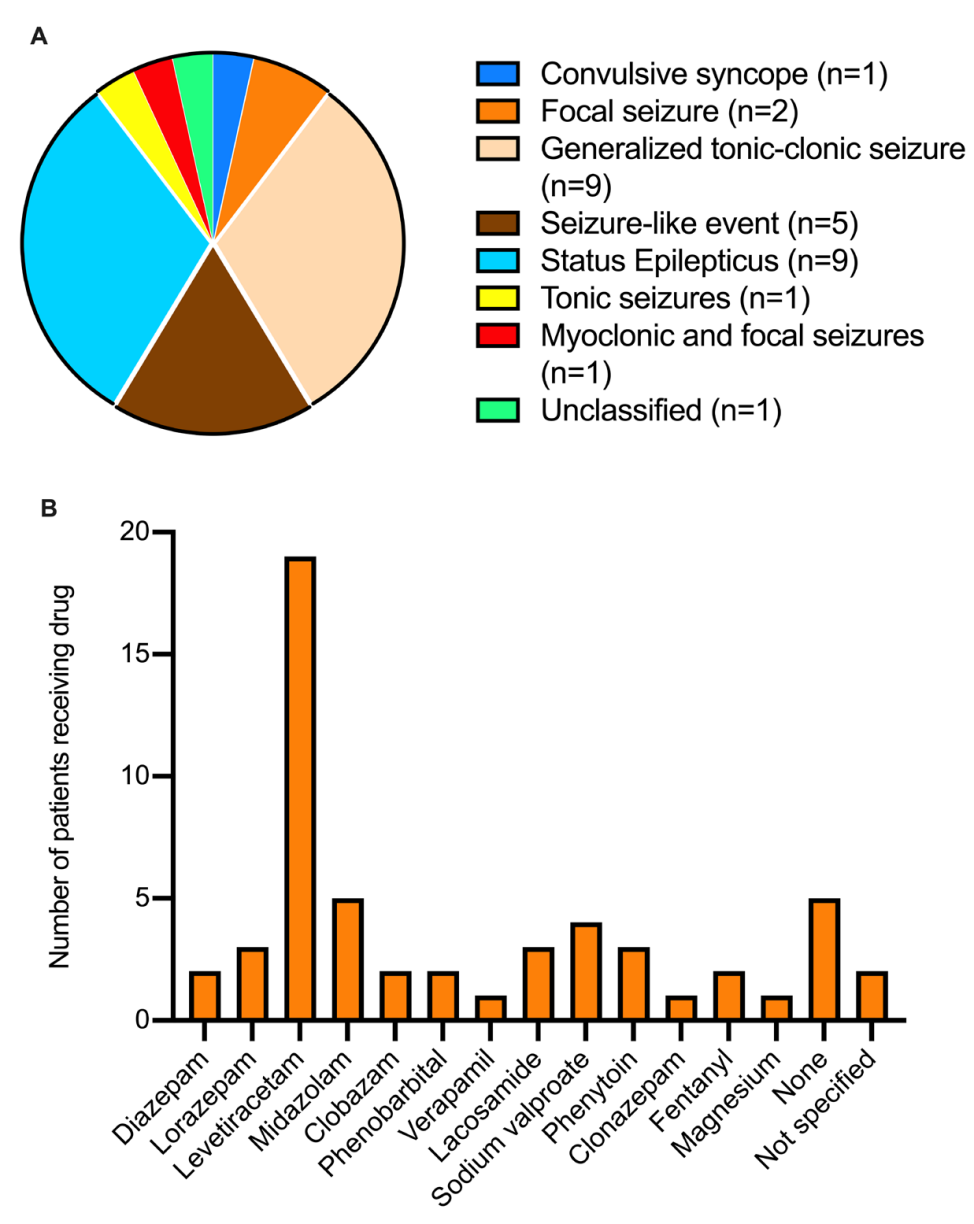
Seizure types ( **A**) and of anti-epilepsy drugs administered ( **B**) in 29 coronavirus disease 2019 (COVID-19) patients with seizure disorders.

The distribution of the types of seizures presented by the patients are summarized in Figure 2a. Most patients presented with either generalized tonic-clonic Seizures (9/29; 31%) or status epilepticus (9/29; 31%). Two patients presented with non-convulsive status epilepticus while one patient suffered refractory status epilepticus. The majority (19/29; 65.5%) of patients received levetiracetam as drug choice or as part of their regimen ( Figure 2b). Moreover, 17.2% (5/29) and 13.8% (4/29) received midazolam and sodium valproate as drug choice or as part of their regimen, respectively, whereas 17.2% (5/29) received no drug therapy ( Figure 2).

### Seizures and patient outcomes

In all, 24.1% (7/29) of patients died, including 9.1% (1/11) of patients with no comorbidities and 33.3% (6/18) of those with ≥1 comorbidity. Moreover, 28.6% (4/14) of patients who received no COVID-19 treatment drugs died whereas 20% (3/15) of those who received COVID-19 treatment drugs died. Overall, 31.0% (9/29) of patients had recurrent seizures of whom 22.22% (2/9) died. None of the patients who received midazolam as drug choice or as part of their regimen developed recurrent seizures whereas 52.6% (10/19) and 25% (1/5) of patients who received levetiracetam and sodium valproate as drug choice or as part of their regimen developed recurrent seizures, respectively ( Table 2). The proportion of patients who received midazolam, levetiracetam, or sodium valproate as drug choice or as part of their regimen who died were 60% (3/5), 26.3% (5/19), and 50% (2/4), respectively. Moreover, none of patients who received no AEDs suffered recurrent seizures or died.

**Table 2.** Outcomes of AED-treated COVID-19 patients experiencing seizures.

AED(s)	Recurrent seizures		Survived	
	Yes (n = 9)	No (n = 20)	Yes (n = 22)	No (n = 7)
**Levetiracetam** (n = 19)	9 (47.37)	10 (52.63)	14 (73.68)	5 (26.31)
**Midazolam** (n = 5)	0 (0)	5 (100)	2 (40)	3 (60)
**Sodium Valproate** (n = 4)	3 (75)	1 (25)	2 (50)	2 (50)
**Other drugs** (n=2)	0 (0)	2 (100)	1 (50)	1 (50)
**No drug** (n=6)	0 (0)	6 (100)	6 (100)	0 (0)

*AED = antiepileptic drug; COVID-19= coronavirus disease 2019*

## Discussion

This systematic review synthesized evidence on the pharmacological management and outcomes in patients with COVID-19 who experienced seizure disorders. Our findings revealed significant heterogeneity in the pharmacological management of seizures in COVID-19 patients. This may be ascribed to the limited knowledge of the pathophysiology of seizures in COVID-19 patients and the lack of evidence-based guidelines. In all, nearly 1 in 4 patients died which may reflect the poor prognosis and clinical challenge in managing these patients. The study also identified status epilepticus and generalized tonic-clonic seizures as the seizure types with highest incidence in COVID-19 patients. Moreover, development of recurrent seizures and mortality appeared to vary according to the AED used.

Researchers have related the incidence of seizures in COVID- 19 patients to factors such as multiple organ failure, hypoxia and severe metabolic and electrolyte changes that may be experienced by these patients ( Asadi-Pooya *et al.*, 2020). A suggested mechanism by ( Nikbakht *et al.*, 2020) associates the incidence of seizures in patients with COVID-19 to increased proinflammatory cytokine (IL-1B, IL-6, TNF-a) levels by microglia/astrocytes in the brain after viral entry through nerve pathways (directly) or ACE2 receptors (indirectly). This results in the elevation of glutamate and aspartate levels, and reduction in levels of Gamma-amino butyric acid (GABA) and disruption of the blood brain barrier (BBB), in the CNS. These effects are notably involved in the pathophysiology of seizures in patients ( Nikbakht *et al.*, 2020).

Evidence from literature suggests poorly-controlled brain inflammation in the pathophysiology of status epilepticus (SE). Elevated levels of proinflammatory cytokines have been identified in the CSF of patients with refractory SE ( Wang & Chen, 2018). Generalized seizure pathogenesis has also been associated with cytokines - TNF-a, IL-1B and IL-6 ( Dede *et al.*, 2017). The increased cytokine levels suggested in COVID-19 may explain the high incidence of status epilepticus and generalized seizures in COVID-19 patients.

This underlying pathological association may indicate that as with other conditions, impairments are best managed by treatment of the underlying disease ( Tinetti & Fried, 2004). In congruence with this, it was observed, that patients who did not receive AEDs but managed for COVID-19 only did not suffer recurrent seizures and survived. However, idiosyncratic resolution of seizures of other patients in the absence of medication therapy was also observed.

The respiratory depressive effect of midazolam may underlie the increased mortality in patients taking midazolam for seizure management identified in this study. COVID-19 can lead to significant hypoxaemia ( Simonson *et al*., 2021) that may be worsened by benzodiazepine- induced respiratory depression which can also cause hypoxaemia and/or hypercapnia ( Gonzalez Castro *et al*., 2017). 

Levetiracetam is one of three drugs identified to have high efficacy and comparable side effect profiles in the management of refractory SE ( Chamberlain *et al.*, 2020; Kapur *et al.*, 2019). Regardless, the benzodiazepines remain first line in SE according to the NICE Guidelines ( Nunes *et al.*, 2012). Levetiracetam is an AED initially approved for partial onset seizure as an adjunct and subsequently as an adjunct in juvenile myoclonic seizures and generalized tonic-clonic seizures in the US. In the European Union, it is used as initial monotherapy in these conditions. With low protein binding and predominant kidney excretion, pharmacokinetic interactions are not a major concern. However, enzyme-inducing AEDs have been shown to cause a reduction in levetiracetam serum levels and promote higher clearance ( Abou-Khalil, 2008). Its good pharmacokinetic interaction profile may warrant its use in COVID-19 cases. The mechanism of action of levetiracetam is via binding to SV2A, a synaptic vesicle protein, which results in the reduction of vesicle release rate of neurotransmitters ( Lynch *et al.*, 2004).

Levetiracetam has been identified to decrease levels of the cytokines, IL-1B, IL-2 and TNF-a ( Himmerich *et al.*, 2013) and may therefore be beneficial in seizure management in COVID-19 patients. However, a study conducted by Li *et al.* (2013) revealed the role of levetiracetam in inhibiting CD8+ T-Lymphocyte function, which is key in protection against viral infections. The study related this effect to the elevated incidence of upper respiratory tract infections in patients receiving levetiracetam. Administration of levetiracetam in COVID-19 patients may therefore be associated with detrimental respiratory effects and may explain the association between increased mortality and levetiracetam administration in COVID-19 patients with seizures as seen in our study. However, further research will be needed in drawing definite conclusions on this association.

The finding of increased mortality in patients with SE is consistent with the general high mortality associated with SE. Status epilepticus has evidently remained a clinical emergency with poor short- and long-term outcomes as well as high morbidity and mortality in the affected population ( Marawar *et al.*, 2018; Stelzer *et al.*, 2015). An acute symptomatic cause such as a CNS infection, as occurs in COVID-19, has been identified as a critical factor related to morbidity. Treatment challenges such as delayed initiation of therapy and lack of effective medication have been identified to be possibly related to higher mortality ( Stelzer *et al.*, 2015).

## Conclusion

The adequacy of antiseizure medication administered may be of concern and there is a need for the establishment of proper seizure management guidelines in COVID-19.

## Study limitation

Our study involved the use of case reports with no controls and a significantly small number of patients.

## Data availability

All data underlying the results are available as part of the article and no additional source data are required.

### Reporting guidelines

DRYAD: PRISMA checklist for ‘Pharmacological management of seizures in patients with COVID-19: a systematic review’. https://doi.org/10.5061/dryad.2jm63xspf.

Data are available under the terms of the Creative Commons Zero "No rights reserved" data waiver (CC0 1.0 Public domain dedication).

## References

[ref-1] Abdulsalam MA Abdulsalam AJ Shehab D : Generalized status epilepticus as a possible manifestation of COVID‐19. *Acta Neurol Scand.* Wiley Online Library,2020;142(4):297–298. 10.1111/ane.13321 32779768 PMC7405327

[ref-2] Abou-Khalil B : Levetiracetam in the treatment of epilepsy. *Neuropsychiatr Dis Treat.* 2008;4(3):507–23. 10.2147/ndt.s2937 18830435 PMC2526377

[ref-3] Asadi-Pooya AA Attar A Moghadami M : Management of COVID-19 in people with epilepsy: drug considerations. *Neurol Sci.* 2020;41(8):2005–2011. 10.1007/s10072-020-04549-5 32594268 PMC7320844

[ref-4] Baig AM Khaleeq A Ali U : Evidence of the COVID-19 Virus Targeting the CNS: Tissue Distribution, Host-Virus Interaction, and Proposed Neurotropic Mechanisms. *ACS Chem Neurosci.* 2020;11(7):995–998. 10.1021/acschemneuro.0c00122 32167747

[ref-5] Balloy G Leclair-Visonneau L Péréon Y : Non-lesional status epilepticus in a patient with coronavirus disease 2019. *Clin Neurophysiol.* 2020;131(8):2059–2061. 10.1016/j.clinph.2020.05.005 32405258 PMC7217773

[ref-6] Bartiromo M Borchi B Botta A : Threatening drug‐drug interaction in a kidney transplant patient with Coronavirus Disease 2019 (COVID‐19). *Transpl Infect Dis.* 2020;22(4):e13286. 10.1111/tid.13286 32279418 PMC7262190

[ref-7] Bernard-Valnet R Pizzarotti B Anichini A : Two patients with acute meningoencephalitis concomitant with SARS-CoV-2 infection. *Eur J Neurol.* 2020;27(9):e43–e44. 10.1111/ene.14298 32383343 PMC7267660

[ref-8] Beyls C Martin N Hermida A : Lopinavir-ritonavir treatment for COVID-19 infection in intensive care unit: risk of bradycardia. *Circ Arrhythm Electrophysiol.* 2020;13(8):e008798. 10.1161/CIRCEP.120.008798 32809882 PMC7446985

[ref-9] Bhatta S Sayed A Ranabhat B : New-onset seizure as the only presentation in a child with COVID-19. *Cureus.* 2020;12(6):e8820. 10.7759/cureus.8820 32742835 PMC7384710

[ref-10] Chamberlain JM Kapur J Shinnar S : Efficacy of levetiracetam, fosphenytoin, and valproate for established status epilepticus by age group (ESETT): a double-blind, responsive-adaptive, randomised controlled trial. *Lancet.* 2020;395(10231):1217–1224. 10.1016/S0140-6736(20)30611-5 32203691 PMC7241415

[ref-11] Chen T Dai Z Mo P : Clinical Characteristics and Outcomes of Older Patients with Coronavirus Disease 2019 (COVID-19) in Wuhan, China: A Single-Centered, Retrospective Study. *J Gerontol A Biol Sci Med Sci.* 2020;75(9):1788–1795. 10.1093/gerona/glaa089 32279081 PMC7184388

[ref-12] Dede F Karadenizli S Özsoy ÖD : The effects of adenosinergic modulation on cytokine levels in a pentylenetetrazole-induced generalized tonic-clonic seizure model. *Neuroimmunomodulation.* 2017;24(1):54–59. 10.1159/000478659 28793299

[ref-13] Desforges M Le Coupanec A Dubeau P : Human coronaviruses and other respiratory viruses: underestimated opportunistic pathogens of the central nervous system? *Viruses.* 2020;12(1):14. 10.3390/v12010014 31861926 PMC7020001

[ref-14] Duong L Xu P Liu A : Meningoencephalitis without respiratory failure in a young female patient with COVID-19 infection in Downtown Los Angeles, early April 2020. *Brain Behav Immun.* 2020;87:33. 10.1016/j.bbi.2020.04.024 32305574 PMC7162766

[ref-15] Elgamasy S Kamel MG Ghozy S : First Case of Focal Epilepsy Associated with SARS‐Coronavirus‐2. *J Med Virol.* 2020;92(10):2238–2242. 10.1002/jmv.26113 32484990 PMC7300744

[ref-16] Emami A Fadakar N Akbari A : Seizure in patients with COVID-19. *Neurol Sci.* 2020;41(11):3057–3061. 10.1007/s10072-020-04731-9 32949289 PMC7501768

[ref-17] Fasano A Cavallieri F Canali E : First motor seizure as presenting symptom of *SARS-CoV-2* infection. *Neurol Sci.* 2020;41(7):1651–1653. 10.1007/s10072-020-04460-z 32417987 PMC7229435

[ref-18] Gaughan M Connolly S Direkze S : Acute new-onset symptomatic seizures in the context of mild COVID-19 infection. *J Neurol.* 2020;1–3. 10.1007/s00415-020-10214-w 32910253 PMC7482052

[ref-19] Gómez-Enjuto S Hernando-Requejo V Lapeña-Motilva J : Verapamil as treatment for refractory status epilepticus secondary to PRES syndrome on a SARS-Cov-2 infected patient. *Seizure.* 2020;80:157–158. 10.1016/j.seizure.2020.06.008 32574838 PMC7275169

[ref-50] Gonzalez Castro LN Mehta JH Brayanov JB : Quantification of respiratory depression during pre-operative administration of midazolam using a non-invasive respiratory volume monitor. *PLoS One.* 2017;12(2):e0172750. 10.1371/journal.pone.0172750 28235069 PMC5325566

[ref-20] Haddad S Tayyar R Risch L : Encephalopathy and seizure activity in a COVID-19 well controlled HIV patient. *IDCases.* 2020;21:e00814. 10.1016/j.idcr.2020.e00814 32426230 PMC7228895

[ref-21] Hepburn M Mullaguri N George P : Acute Symptomatic Seizures in Critically Ill Patients with COVID-19: Is There an Association? *Neurocrit Care.* 2021;34(1):139–143. 10.1007/s12028-020-01006-1 32462412 PMC7253233

[ref-22] Himmerich H Bartsch S Hamer H : Impact of mood stabilizers and antiepileptic drugs on cytokine production *in-vitro*. *J Psychiatr Res.* 2013;47(11):1751–1759. 10.1016/j.jpsychires.2013.07.026 23978396

[ref-23] Kapur J Elm J Chamberlain JM : Randomized trial of three anticonvulsant medications for status epilepticus. *N Engl J Med.* 2019;381(22):2103–2113. 10.1056/NEJMoa1905795 31774955 PMC7098487

[ref-24] Karambelkar PV Rojulpote CS Saeed F : The Neurological Manifestations of COVID-19–A Case Series. *J Clin Transl Res.* 2020;6(4):187–189. 33501389 PMC7821746

[ref-25] Lan J Ge J Yu J : Structure of the SARS-CoV-2 spike receptor-binding domain bound to the ACE2 receptor. *Nature.* 2020;581(7807):215–220. 10.1038/s41586-020-2180-5 32225176

[ref-27] Lau KK Yu WC Chu CM : Possible central nervous system infection by SARS coronavirus. *Emerg Infect Dis.* 2004;10(2):342–4. 10.3201/eid1002.030638 15030709 PMC3322928

[ref-28] Li G Nowak M Bauer S : Levetiracetam but not valproate inhibits function of CD8 ^+^ T lymphocytes. *Seizure.* 2013;22(6):462–466. 10.1016/j.seizure.2013.03.006 23639870

[ref-29] Logmin K Karam M Schichel T : Non-epileptic seizures in autonomic dysfunction as the initial symptom of COVID-19. *J Neurol.* 2020;267(9):2490–2491. 10.1007/s00415-020-09904-2 32458194 PMC7249974

[ref-30] Lynch BA Lambeng N Nocka K : The synaptic vesicle protein SV2A is the binding site for the antiepileptic drug levetiracetam. *Proc Natl Acad Sci U S A.* 2004;101(26):9861–9866. 10.1073/pnas.0308208101 15210974 PMC470764

[ref-31] Mao L Jin H Wang M : Neurologic manifestations of hospitalized patients with coronavirus disease 2019 in Wuhan, China. *JAMA Neurol.* 2020;77(6):683–690. 10.1001/jamaneurol.2020.1127 32275288 PMC7149362

[ref-32] Marawar R Basha M Mahulikar A : Updates in refractory status epilepticus. *Crit Care Res Pract.* 2018;2018:9768949. 10.1155/2018/9768949 29854452 PMC5964484

[ref-33] Moher D Liberati A Tetzlaff J : Preferred reporting items for systematic reviews and meta-analyses: the PRISMA statement. *Int J Surg.* 2010;8(5):336–341. 10.1016/j.ijsu.2010.02.007 20171303

[ref-34] Moriguchi T Harii N Goto J : A first case of meningitis/encephalitis associated with SARS-Coronavirus-2. *Int J Infect Dis.* 2020;94:55–58. 10.1016/j.ijid.2020.03.062 32251791 PMC7195378

[ref-51] Murad MH Sultan S Haffar S : Methodological quality and synthesis of case series and case reports. *BMJ Evid Based Med.* 2018;23:60–63. 10.1136/bmjebm-2017-110853 29420178 PMC6234235

[ref-35] Nikbakht F Mohammadkhanizadeh A Mohammadi E : How does the COVID-19 cause seizure and epilepsy in patients? The potential mechanisms. *Mult Scler Relat Disord.* 2020;46:102535. 10.1016/j.msard.2020.102535 33010584 PMC7521932

[ref-36] Nunes VD Sawyer L Neilson J : Diagnosis and management of the epilepsies in adults and children: summary of updated NICE guidance. *BMJ.* 2012;344:e281. 10.1136/bmj.e281 22282528

[ref-37] Page MJ McKenzie JE Bossuyt PM : The PRISMA 2020 statement: an updated guideline for reporting systematic reviews. *BMJ.* 2021;372:n71. 10.1136/bmj.n71 33782057 PMC8005924

[ref-52] Simonson TS Baker TL Banzett RB : Silent hypoxaemia in COVID-19 patients. *J Physiol.* 2021;599(4):1057–65. 10.1113/JP280769 33347610 PMC7902403

[ref-38] Sohal S Mansur M : COVID-19 Presenting with Seizures. *IDCases.* 2020;20:e00782. 10.1016/j.idcr.2020.e00782 32363146 PMC7194035

[ref-39] Stelzer FG Bustamante GdO Sander H : Short-term mortality and prognostic factors related to status epilepticus. *Arq Neuropsiquiatr.* 2015;73(8):670–675. 10.1590/0004-282X20150082 26222358

[ref-53] Tinetti ME Fried T : The end of the disease era. *Am J Med.* 2004;116(3):179–85. 10.1016/j.amjmed.2003.09.031 14749162

[ref-40] Wang M Chen Y : Inflammation: a network in the pathogenesis of status epilepticus. *Front Mol Neurosci.* 2018;11:341. 10.3389/fnmol.2018.00341 30344475 PMC6182087

[ref-41] Zanin L Saraceno G Panciani PP : SARS-CoV-2 can induce brain and spine demyelinating lesions. *Acta Neurochir (Wien).* 2020;162(7):1491–1494. 10.1007/s00701-020-04374-x 32367205 PMC7197630

