# Supplementary material for: Pharmacological management of seizures in patients with COVID-19: a systematic review
Source: AAS Open Res. 2021 Nov 23;4:29. Originally published 2021 Jun 9. [Version 2] doi: 10.12688/aasopenres.13224.2 (PMC9648370; doi:10.12688/aasopenres.13224.2)
Supplement: Peer review history

# Peer review: Reviewer response for version 2

**DOI:** 10.21956/aasopenres.14455.r28988

**Published:** 2022-05-06

**Authors:** Ella A. Kasanga, Anthony Oppong-Gyebi

**Affiliations:** 1 Center for Healthy Aging, University of North Texas Health Science Center, Fort Worth, TX, USA; 2 Biogen Inc., Cambridge, MA, USA

The authors have fully addressed the comments and suggestions provided in the initial review.

Are the rationale for, and objectives of, the Systematic Review clearly stated?

Partly

Is the statistical analysis and its interpretation appropriate?

Yes

Are sufficient details of the methods and analysis provided to allow replication by others?

Yes

Are the conclusions drawn adequately supported by the results presented in the review?

Partly

Reviewer Expertise:

Parkinson's disease, stroke, neurological disorders, neuropharmacology

We confirm that we have read this submission and believe that we have an appropriate level of expertise to confirm that it is of an acceptable scientific standard.

# Peer review: Reviewer response for version 1

**DOI:** 10.21956/aasopenres.14344.r28657

**Published:** 2021-07-26

**Authors:** Thomas A. Tagoe

**Affiliations:** 1 Department of Physiology, UG Medical School, College of Health Sciences, University of Ghana, Accra, Ghana

The authors present here a systemic review of case reports on the incidence of seizures among patients who were hospitalised due to COVID-19 infections. The reports include those published between January to December 2020. The databases that were searched as well as the search string used is provided to allow reproduction of their work. The authors identify 67 articles which was narrowed down to 19. Based on these reports, the frequency of specific anti epileptic drugs used in case management is clearly reported as well as the type of seizures and patient outcomes. Studies that reported on patients with a history of seizures were excluded. Only cases of seizure complication for which a temporal or causal relationship with COVID-19 infection could be determined were included. This contributed towards focusing the scope of the review. The authors do a good job of pulling out key pieces of information from each report and discuss the potential pathways by which AEDs could overlap with COVID-19. Based on the reports presented as part of the review, the authors conclude that AEDs could potentially be worsening patient outcomes. Although the review is scientifically sound, there are a few outstanding points which could further strengthen the review if addressed.

1. Figure 1 shows that 48 out of 67 retrieved records were excluded. Details on the common reasons for exclusion among the 48 cases that were cut will be useful. 
2. According to the methodology, data extracted included "...frequency and prevalence of seizures, electroencephalogram (EEG), neuroimaging and/or other laboratory investigations associated with other neuropathological symptoms,...". However this information is not included in any of the tables or results presented.
3. Under the study characteristics, there is no basis given for judging case reports as "fair", "good" or "excellent".
4. The labelling of the flow diagram in figure 1 could be done vertically rather than horizontally, purely for aesthetic reasons and better reading.
5. Information on seizure history is absent for some of the reported cases (9 and 10). Considering that this was a critical point in selection of cases, it will be great to know if this information was absent from the primary reports or erroneously left out of the review. 
6. The data in this review clearly shows that all the patients who did not receive AEDs (n = 6), did not suffer recurrent seizures and also survived. This is a curious finding which could have been further explored in the discussion.  
7. Based on the mechanism of action of levetiracetam, the authors propose that "Administration of levetiracetam in COVID-19 patients may therefore be associated detrimental respiratory effects and may explain the association between increased mortality and levetiracetam administration in COVID-19 patients with seizures as seen in our study". Although this is sound, patients that received levetiracetam also showed the highest survival rates among all the patients who received AEDs. A discussion around another AED with a different mechanism of action from levetiracetam and  worse survival rates would have enriched the review. 

Are the rationale for, and objectives of, the Systematic Review clearly stated?

Yes

Is the statistical analysis and its interpretation appropriate?

Not applicable

Are sufficient details of the methods and analysis provided to allow replication by others?

Yes

Are the conclusions drawn adequately supported by the results presented in the review?

Yes

Reviewer Expertise:

Epilepsy, Plasticity, Learning and Memory

I confirm that I have read this submission and believe that I have an appropriate level of expertise to confirm that it is of an acceptable scientific standard.

# Response

**Published:** 2021-11-05

**Authors:** Priscilla Mante

**Affiliations:** Kwame Nkrumah University of Science and Technology, Ghana

1. Figure 1 shows that 48 out of 67 retrieved records were excluded. Details on the common reasons for exclusion among the 48 cases that were cut will be useful. 

**Answer: **The authors have provided information on the inclusion/exclusion criteria in the methods section. The 48 records excluded did not meet the strict inclusion criteria.

 2. According to the methodology, data extracted included "...frequency and prevalence of seizures, electroencephalogram (EEG), neuroimaging and/or other laboratory investigations associated with other neuropathological symptoms,...". However this information is not included in any of the tables or results presented.

**Answer: **This information, where available and relevant to the study and findings, were added to the outcome section of Table 1.

 3. Under the study characteristics, there is no basis given for judging case reports as "fair", "good" or "excellent".

**Answer:** The Murad tool was used to evaluate the studies for methodological quality and risk of bias. Studies were considered as “poor,” “moderate/fair,” or “good/excellent” quality when 3 or fewer, 4, or 5 of

 the criteria were fulfilled, respectively. This criterion has been updated in the manuscript.

 4. The labelling of the flow diagram in figure 1 could be done vertically rather than horizontally, purely for aesthetic reasons and better reading.

**Answer: **The authors would appreciate further clarification on this comment.

 5. Information on seizure history is absent for some of the reported cases (9 and 10). Considering that this was a critical point in selection of cases, it will be great to know if this information was absent from the primary reports or erroneously left out of the review. 

**Answer:** The seizure histories of patients 9 and 10 were not reported and negative respectively; the table has been updated accordingly.

 6. The data in this review clearly shows that all the patients who did not receive AEDs (n = 6), did not suffer recurrent seizures and also survived. This is a curious finding which could have been further explored in the discussion.

**Answer:** The discussion has been updated accordingly. 

 7. Based on the mechanism of action of levetiracetam, the authors propose that "Administration of levetiracetam in COVID-19 patients may therefore be associated detrimental respiratory effects and may explain the association between increased mortality and levetiracetam administration in COVID-19 patients with seizures as seen in our study". Although this is sound, patients that received levetiracetam also showed the highest survival rates among all the patients who received AEDs. A discussion around another AED with a different mechanism of action from levetiracetam and  worse survival rates would have enriched the review. 

**Answer:** The discussion has been updated accordingly.

# Peer review: Reviewer response for version 1

**DOI:** 10.21956/aasopenres.14344.r28660

**Published:** 2021-07-06

**Authors:** Ella A. Kasanga, Anthony Oppong-Gyebi

**Affiliations:** 1 Center for Healthy Aging, University of North Texas Health Science Center, Fort Worth, TX, USA; 2 Biogen Inc., Cambridge, MA, USA

This is an interesting review on reported cases of seizure disorders in COVID-19 cases in 2020, the diverse pharmacological agents used and outcomes on mortality. The authors shed light on the limitations of this review as there are not a lot of reported cases within the timeline of the investigation. In our opinion, the authors should consider the following highlighted points to expound on the findings from their review.

Major Comments

1. In reading the introduction, the authors state the information provided by this review. However, it is still unclear what unmet need this review is aiming to answer. Providing more clarification (at least a sentence or two) on the purpose of this review, in our opinion, will underscore the relevance of the review.
2. What were the criteria for selecting the 4 electronic literature search platforms used for this review? Could more information be garnered from these additional search platforms - the Cochrane Library, Web of Science Core Collection, CENTRAL, and clinicaltrials.gov?
3. What was the rubric for the grading system for the reports (fair quality vs good/excellent quality)?
4. There are a number of inconsistencies in the patient and publication counts. Specifically,
  1. It is stated that there are 19 studies/references but Table 1 only shows 18 references.
  2. Under patient characteristics, it is stated that the patients were from 9 countries, however, there are 10 countries listed.
  3. The patient count from the patient characteristics section adds up to 29, however, the table only shows data for 28 patients.
5. In table 1, for patients 9 and 10, are the seizure histories not reported or negative? Please clarify.
6. The authors stated that there is significant heterogeneity in the pharmacological management of seizures in COVID-19. Was that conclusion based on any inferential statistics run or it was based on the observation from the descriptive statistics?
7. In patient 16, Moriguchi *et al*. ^1^ indicated that the patient received Laninamivir. Is this the same as Laminavir indicated in Table 1? Why is there an asterisk on Laminavir (no information is provided on the use of the asterisk in the table legend)?
8. The conclusion of the review article could be expounded a little bit further. The authors stated that ‘The adequacy of anti-seizure medication administered may be of concern’. How did they come up with this conclusion?
9. In the title of this review, the authors refer to the pharmacological management of seizures. However, the term, 'pharmacological management' does not just entail the choice of pharmacological agent (which is the highlight of this review), but also the duration of therapy, dose, possible side effects, and other aspects. Thus, in our opinion, the title does not fully represent the content of the review.

Minor Comments

1. In the co-morbidities column for table 1, different terms have been used to signify no comorbidities – no, nil of note, and none stated. The authors can consider using one term for consistency.
2. In table 1, the seizure description for patients 7 & 8 are incomplete (as compared to patients 12, 13 and 14).
3. Please define ROSC (as used for patient 19 in table 1).
4. There are words omitted in the discussion section, for instance:
  1. This results in the elevation ***of*** glutamate and aspartate levels…………………….
  2. Administration of levetiracetam in COVID-19 patients may therefore be associated ***with*** detrimental……

Are the rationale for, and objectives of, the Systematic Review clearly stated?

Partly

Is the statistical analysis and its interpretation appropriate?

Yes

Are sufficient details of the methods and analysis provided to allow replication by others?

Yes

Are the conclusions drawn adequately supported by the results presented in the review?

Partly

Reviewer Expertise:

Parkinson's disease, stroke, neurological disorders, neuropharmacology

We confirm that we have read this submission and believe that we have an appropriate level of expertise to confirm that it is of an acceptable scientific standard, however we have significant reservations, as outlined above.

## References

[1] : A first case of meningitis/encephalitis associated with SARS-Coronavirus-2. *Int J Infect Dis* .2020;94: 10.1016/j.ijid.2020.03.062 55-58 10.1016/j.ijid.2020.03.062 32251791PMC7195378

# Response

**Published:** 2021-11-05

**Authors:** Priscilla Mante

**Affiliations:** Kwame Nkrumah University of Science and Technology, Ghana

Major Comments:

 1. In reading the introduction, the authors state the information provided by this review. However, it is still unclear what unmet need this review is aiming to answer. Providing more clarification (at least a sentence or two) on the purpose of this review, in our opinion, will underscore the relevance of the review.Answer:

**Answer: **The introduction has been updated accordingly.

 2. What were the criteria for selecting the 4 electronic literature search platforms used for this review? Could more information be garnered from these additional search platforms - the Cochrane Library, Web of Science Core Collection, CENTRAL, and clinicaltrials.gov?

**Answer: **The four search platforms were made the focus of the search after preliminary search for such cases. Given that the review was focused on case reports due to the scarcity of such cases, majority of the databases stated by reviewers were not useful.

 3. What was the rubric for the grading system for the reports (fair quality vs good/excellent quality)?Answer: The Murad tool was used to evaluate the studies for **methodological quality** and risk of bias. Studies were considered as “poor,” “moderate,” or “good/excellent” quality when 3 or fewer, 4, or 5 of

 the criteria were fulfilled, respectively.

**Answer: **This criterion has been updated in the manuscript.

 4. There are a number of inconsistencies in the patient and publication counts. Specifically,

 It is stated that there are 19 studies/references but Table 1 only shows 18 references.

**Answer: **This has been corrected.

 Under patient characteristics, it is stated that the patients were from 9 countries, however, there are 10 countries listed.

**Answer:** This has been corrected.

 The patient count from the patient characteristics section adds up to 29, however, the table only shows data for 28 patients.

**Answer: **Table 1 does show data for 29 patients and not 28, in accordance with the patient count from the patient characteristics.

 5. In table 1, for patients 9 and 10, are the seizure histories not reported or negative? Please clarify.

**Answer:** The seizure histories of patients 9 and 10 were not reported and negative respectively; the table has been updated accordingly.

 6. The authors stated that there is significant heterogeneity in the pharmacological management of seizures in COVID-19. Was that conclusion based on any inferential statistics run or it was based on the observation from the descriptive statistics?

**Answer:** This conclusion was based on the observation from the descriptive characteristics.

 7. In patient 16, Moriguchi *et al*. ^1^ indicated that the patient received Laninamivir. Is this the same as Laminavir indicated in Table 1? Why is there an asterisk on Laminavir (no information is provided on the use of the asterisk in the table legend)?

**Answer:** The study reported the use of Laninamivir. This has been made consistent throughout the manuscript. The asterisk was used to indicate the ongoing nature of the case. It has however been removed from the table.

 8. The conclusion of the review article could be expounded a little bit further. The authors stated that ‘The adequacy of anti-seizure medication administered may be of concern’. How did they come up with this conclusion?

**Answer: **Based on the information available, adequacy of antiseizure medications used may still be in question as the results of the study. Provide no concrete conclusions on the advantage of specific anti-seizure drugs.

 9. In the title of this review, the authors refer to the pharmacological management of seizures. However, the term, 'pharmacological management' does not just entail the choice of pharmacological agent (which is the highlight of this review), but also the duration of therapy, dose, possible side effects, and other aspects. Thus, in our opinion, the title does not fully represent the content of the review.

**Answer:** The term ‘pharmacological management’ may also simply describe the use of drug therapy for management of conditions and as such, may have emphasis laid on medication choice

Minor Comments

 1. In the co-morbidities column for table 1, different terms have been used to signify no comorbidities – no, nil of note, and none stated. The authors can consider using one term for consistency.

**Answer:** Authors agree that the use of ‘nil of note’ and ‘no’ connote the same thing and has all been changed to ‘nil of note’ for consistency. However, the use of ‘none stated’ is to suggest that the reference provided no such information and as such, does not suggest the absolute absence of any comorbidity.

 2. In table 1, the seizure description for patients 7 & 8 are incomplete (as compared to patients 12, 13 and 14).

**Answer:** This has been corrected.

 3. Please define ROSC (as used for patient 19 in table 1).

**Answer:** ROSC has been defined in table 1.

 4. There are words omitted in the discussion section, for instance:

 This results in the elevation ***of *** glutamate and aspartate levels…………………….

**Answer:** This has been corrected.

 Administration of levetiracetam in COVID-19 patients may therefore be associated ***with *** detrimental……

**Answer: **This has been corrected.
